# Serologic Detection of Anti Toxoplasma gondii Infection in Diabetic Patients

**DOI:** 10.5812/ircmj.5303

**Published:** 2013-08-05

**Authors:** Shahnaz Shirbazou, Ali Delpisheh, Rahim Mokhetari, Ghafor Tavakoli

**Affiliations:** 1 Department of Parasitology, Baqiyatallah University of Medical Sciences, Tehran, IR Iran; 2 Department of Clinical Epidemiology and Prevention of Psychosocial Injuries, Research Centre, Ilam University of Medical Sciences, Ilam, IR Iran; 3 Department of Medical Technology, Shahid Beheshti University of Medical Sciences, Tehran, IR Iran

**Keywords:** Diabetes, Infection, Serum, Toxoplasma gondii

## Abstract

**Background:**

Toxoplasmosis is caused by the *Toxoplasma* *gondii* parasite. The parasite is intracellular and can result in severe complications leading to death in immuno-deficient patients in particular. Diabetes is an important factor that increases susceptibility and risk of various infections in the host.

**Objectives:**

The present study focused on the serologic detection of *Toxoplasma gondii* infection in diabetic patients.

**Materials and Methods:**

Through a case-control study, overall 184 serum samples including 91 from diabetic cases and 93 from healthy non-diabetic controls were investigated. Cases and controls were matched for age and gender. Serum samples were tested for sugar by an enzymatic method, and IgG antibodies were tested against *Toxoplasma gondii* by ELISA method.

**Results:**

The prevalence of IgG antibodies against *Toxoplasma gondii* in diabetic patients and healthy controls were 60.43% and 38% respectively. Risk of toxoplasmosis infection in diabetic patients with was two folds higher than healthy controls (RR = 2.21, 95% CI; 1.6 – 3.7, P = 0.001).

**Conclusions:**

Diabetes may be caused by *Toxoplasma gondii*. Presence of *T. gondii* in the pancreas at the same time could directly undermines the pancreas cells. When β cells are destroyed, insulin secretion would then be affected. Probably the destruction of *T. gondii* affects nervous system and damages pancreatic cells leading to increased risk of diabetes.

## 1. Background

Toxoplasmosis is a disease caused by the protozoan parasite *Toxoplasma gondii.* Up to one third of the world's human population is estimated to be carrying a *Toxoplasma* infection and the life cycle of this parasite plays a huge role in its easy transmission (1). Only a minority develop serious clinical disease, such as congenital or cerebral toxoplasmosis is associated with an immature and a deficient immunity. So the parasite can be considered as the major cause of central nervous system infections in patients with congenital toxoplasmosis and AIDS (2, 3). The important role played by CD8 T cells in control of *Toxoplasma gondii* infection (4). Immune protection against many intracellular pathogens including viruses, bacteria and protozoa is provided by robust CD8 T-cell responses. Naïve CD8 T cells are found in lymphoid tissues where, after infection, they encounter an antigen-presenting cell (APC) (5). The APC presents pathogen-derived antigens and provides the appropriate signals to T cells to cause their activation. This activation leads to the proliferation, differentiation, and acquisition of effectors functions of the antigen-specific CD8 T cell. Activated antigen-specific CD8 T-cell effectors functions include secretion of cytokines IFNγ and TNFα and cytotoxicity, which promote further development of adaptive immunity and control pathogens (6-8).

Effects of Toxoplasma gondii on central nervous system has been defined by multiple foci of enlarging necrosis and microglial nodules (9).The immune-histo-chemical studies have investigated the distribution of lesions in verity of animal tissues that are experimentally inoculated with *T. gondii* (10, 11). During acute phase of toxoplasmosis, tissue necrosis may be found in various organs including lung, liver, spleen, heart and pancreas (12). In the chronic infection, necrosis always often found in brain and eye. Immuno-histo-chemical techniques have already been used to investigate the distribution of lesions and protozoa in young piglets that were experimentally inoculated with *T. gondii*. According to WHO; diabetes is a chronic disease that occurs when the pancreas does not produce enough insulin, or when the body cannot effectively use the insulin it produces Hyperglycaemia, or raised blood sugar, is a common effect of uncontrolled diabetes and over time leads to serious damage to many of the body's systems, especially the nerves and blood vessels (13).

Diabetes increases the host’s sensitivity and risk of susceptibility to various infections (14). Based on statistics, the number of people diagnosed with diabetes was in excess of 2.5 million within the age range of 25 to 64 in 2007 (15). It is predicted that 366 million would suffer from diabetes around the world in 2030 (16). The present study shows correlation diabetes and toxoplasmosis. Currently, the levels of IgG and IgM antibodies in serum can easily be measured using available techniques (17). Serologic methods are techniques used to measure the level of infection to *Toxoplasmasis* in humans and animals. Amongst these methods, the most common techniques are ELISA and IFA or indirect immuno-florescence (18).

## 2. Objectives

The present study aimed to investigate the effects of *Toxoplamasis* on uncontrolled diabetes and on the level of outbreak of infection in patients, depending on age, with differing immune systems and dietary habits. Hence, the level of IgG antibody against *Toxoplasma gondii* in the serum of diabetic patients is measured.

## 3. Materials and Methods

Through a case-control study, overall 184 serum samples including 91 from diabetic patients and the remaining 93 from healthy non-diabetic controls were investigated. Cases and controls were matched for age and gender. Once sampling was performed, the level of glucose (FBS) was read employing an enzymatic method. In this method, 10 landa serum and 1ml of designated solution was mixed and left for 10 minutes at 37 °C Benmary to be incubated. Then the results were read using Spectrophotometer at wavelength 545nm. Serums were then kept at -20 °C for ELISA testing. Finally, the level of IgG antibody against *Toxoplasma gondii* was measured using the ELISA method and an IgG *Toxoplasma Gondii* kit provided by the Pishtaz Teb Zaman Co. with 100% sensitivity and 99% specificity. The proposal of the present study was approved by the Baghiyatallah University Ethical Committee.

## 4. Results

The abundance distribution for 184 studied samples is demonstrated in Table 1. A positive IgG *Toxoplasmosis *was observed in 60.43% and 38.7% of diabetic cases and health controls respectively (Table 1). Overall, 54.54% and 45.46% of the diabetic categories were females and males respectively. 

**Table 1.** Abundance Distribution of *Toxoplasmosis* Cases

Category	No	*+ Toxoplasmosis*, No. (%)
**Diabetic Cases**	91	55 (60.43)
**Healthy Controls**	93	36 (38.7)
**Total**	184	91 (49.6)

It is found that 24 patients of the diabetic category had glucose levels of 200 – 300 mg/dl which is perceived to be more abundant than the other 2 glucose levels, i.e. 140 – 200 and 300 – 400 mg/dl (Table 2). 

**Table 2.** Glucose Level (FBS) and *Toxoplasmosis* Infection

Glucose levels (Mg/dl)	+ *Toxoplasmosis*, No. (%)
**140-200**	18 (32.7)
**200-300**	24 (43.6)
**300-400**	13 (23.6)
**Total**	55 (100)

Risk of toxoplasmosis infection in diabetic patients with was 2 folds higher than healthy controls (RR = 2.21, 95 CI; 1.6 – 3.7, P = 0.001) (Table 3). 

**Table 3.** Proportional Distribution of *Toxoplasmosis* between Diabetic and Non-Diabetic People

*Toxoplasmosis*	Diabetic Cases, No.	Non-diabetic Controls, No.
**Positive**	55	38
**Negative**	36	55
**Total**	91	93
**Risk estimation**	(RR = 2.21, 95 CI; 1.6 – 3.7, P = 0.001)	

## 5. Discussions

This investigation was carried out with a density of more than 1.20 (units). Overall, 60.43% of diabetic patients and only 38.7% of non-diabetic controls were positive for the *Toxoplasmosis gondii* antibody. Therefore, these findings suggest that *Toxoplasmosis* patients are more susceptible to be diabetics than those without. Destruction of the pancreas occurs in three phases of *Toxoplasma gondii*:

1. Hyperactive phase (hyper-period) in which β-cell destruction of nerve cells and less interference in the insect in a hyperactive state of the pancreas, insulin secretion is sometimes excessive, often resulting in low blood sugar, or a too low blood sugar, this stage is often during adolescence.

2. Disordered phase (compensatory phase), in which neurons and pancreatic β-cells have a considerable amount of damage, under normal circumstances, secretion of insulin will be inadequate, the body will start the compensatory function. Thus, this phase of insulin secretion over time, when few in the disordered state.

3. Decline phase (recession), in which nerve cells and β-cells destruction of more compensatory function reached its limits (19).

A recent experimental study has described that pancreatitis due to Toxoplasmosis in cats. *Toxoplasma Gondii & Amphimerus Pseudofelineus* are microbial agents associated with the disease (20). A stimulative effect of insulin on *Toxoplasma gondii* replication in cells (in vitro) has recently been reported. The number of tachyzoites increased rapidly, with insulin concentrations of between 10^–2^ and 10^–1^ μg/ml (21). Several studies have reported diabetes insipitus in particular (22-24), and other diseases such as Alzheimer (25) in patient with congenital toxoplasmosis. The tissue necrosis in pancreas during acute toxoplasmosis has also been reported (26).

The present study is limited by confounding factors associated with diabetes mellitus. We have been trying to reduce confounders by epidemiological approaches such as individual matching and by setting inclusion and exclusion criteria according to latest definitions of diabetes by the World Health Organization. Thus, *Toxoplasmosis* has also been implicated as a possible contributing factor in chronic pancreatitis also the inadequacy of insulin secretion, sustained blood and increased urine sugar. By further destruction of *Toxoplasma gondii* series of complications such as blindness, diabetic foot, coronary heart disease, and hypertension are expected.

## References

[1] Center for Disease Control. (2009). Laboratory Identification of Parasites of Public Health Concern.. http://www.dpd.cdc.gov/dpdx/HTML/Toxoplasmosis.htm.

[2] Luft BenjaminJ, Remington JackS (1992). Toxoplasmic Encephalitis in AIDS.. Clin Infect Dis..

[3] Porter SB, Sande MA (1992). Toxoplasmosis of the central nervous system in the acquired immunodeficiency syndrome.. N Engl J Med..

[4] Gigley JasonP, Bhadra Rajarshi, Khan ImtiazA (2011). CD8 T Cells and Toxoplasma gondii: A New Paradigm.. J Parasitol Res..

[5] Williams MA, Bevan MJ (2007). Effector and memory CTL differentiation.. Annu Rev Immunol..

[6] Mescher MF, Curtsinger JM, Agarwal P, Casey KA, Gerner M, Hammerbeck CD (2006). Signals required for programming effector and memory development by CD8+ T cells.. Immunol Rev..

[7] Sharpe AH (2009). Mechanisms of costimulation.. Immunol Rev..

[8] Gazzinelli R, Xu Y, Hieny S, Cheever A, Sher A (1992). Simultaneous depletion of CD4+ and CD8+ T lymphocytes is required to reactivate chronic infection with Toxoplasma gondii.. J Immunol..

[9] Montoya JG, Liesenfeld O (2004). Toxoplasmosis.. Lancet..

[10] Banales P, Yamada M, Narita M, Shimura K, Nakamura K (2006). Immunohistochemical distribution of protozoa in experimental porcine toxoplasmosis.. JARQ..

[11] Dubey JP, Hedstrom O, Machado CR, Osborn KG (1991). Disseminated Toxoplasmosis in a Captive Koala.. J Zoo Wildlife Med..

[12] Dubey JP (1996). Infectivity and pathogenicity of Toxoplasma gondii oocysts for cats.. J Parasitol..

[13] World Health Organization. (2011). Diabetes Programme.. http://www.who.int/diabetes/en/.

[14] Joshi Nirmal, Caputo GregoryM, Weitekamp MichaelR, Karchmer AW (1999). Infections in Patients with Diabetes Mellitus.. New Eng J Med..

[15] Esteghamati A, Meysamie A, Khalilzadeh O, Rashidi A, Haghazali M, Asgari F (2009). Third national Surveillance of Risk Factors of Non-Communicable Diseases (SuRFNCD-2007) in Iran: methods and results on prevalence of diabetes, hypertension, obesity, central obesity, and dyslipidemia.. BMC Public Health..

[16] Wild S, Roglic G, Green A, Sicree R, King H (2004). Global prevalence of diabetes: estimates for the year 2000 and projections for 2030.. Diabetes Care..

[17] Wilson M, Remington JS, Clavet C, Varney G, Press C, Ware D (1997). Evaluation of six commercial kits for detection of human immunoglobulin M antibodies to Toxoplasma gondii. The FDA Toxoplasmosis Ad Hoc Working Group.. J Clin Microbiol..

[18] Saabi IS (1997). Parastic Diseases in Iran..

[19] (2012). What Are The Causes For Diabetes?. http://www.Blurtit.com.

[20] (2012). Pancreatitis in Cats.. http://www.cat-health-guide.org/pancreatitis-in-cats.html.

[21] Zhu S, Lai DH, Li SQ, Lun ZR (2006). Stimulative effects of insulin on Toxoplasma gondii replication in 3T3-L1 cells.. Cell Biol Int..

[22] Karadag A, Erdeve O, Atasay B, Arsan S, Deda G, Ince E (2006). Isolated central diabetes insipidus in a newborn with congenital toxoplasmosis.. J Pediatr Endocrinol Metab..

[23] Oygur N, Yilmaz G, Ozkaynak C, Guven AG (1998). Central diabetes insipitus in a patient with congenital toxoplasmosis.. Am J Perinatol..

[24] Yamakawa Rumi, Yamashita Yushiro, Yano Akihiko, Morita Jun, Kato Hirohisa (1996). Congenital toxoplasmosis complicated by central diabetes insipidus in an infant with down syndrome.. Brain and Development..

[25] Kusbeci OY, Miman O, Yaman M, Aktepe OC, Yazar S (2011). Could Toxoplasma gondii have any role in Alzheimer disease?. Alzheimer Dis Assoc Disord..

[26] Waree P (2008). Toxoplasmosis: Pathogenesis and immune response.. Thammasat Med J..

